# Burden of Common Mental Disorders in Ulcerative Colitis and Irritable Bowel Syndrome Patients: An Analysis of Risk Factors

**DOI:** 10.3390/jcm14020499

**Published:** 2025-01-14

**Authors:** Danusia Onisor, Calin Avram, Florina Ruta, Olga Brusnic, Alina Boeriu, Mircea Stoian, Adrian Boicean, Maria Sasaran

**Affiliations:** 1 Department of Internal Medicine VII, George Emil Palade University of Medicine, Pharmacy, Science and Technology of Targu Mures, Gheorghe Marinescu Street No. 38, 540136 Targu Mures, Romania; danusia.onisor@umfst.ro (D.O.); olga.brusnic@umfst.ro (O.B.); alina.boeriu@umfst.ro (A.B.); 2 Department of Medical Informatics and Biostatistics, George Emil Palade University of Medicine, Pharmacy, Science and Technology of Targu Mures, Gheorghe Marinescu Street No. 38, 540136 Targu Mures, Romania; 3 Department Community Nutrition and Food Safety, Faculty of General Medicine, “George Emil Palade” University of Medicine, Pharmacy, Science, and Technology of Targu Mures, 540142 Targu Mures, Romania; 4 Department of Anesthesiology and Intensive Care, George Emil Palade University of Medicine, Pharmacy, Sciences and Technology of Targu Mures, 540139 Targu Mures, Romania; mircea.stoian@umfst.ro; 5 Faculty of Medicine, Lucian Blaga University of Sibiu, 550169 Sibiu, Romania; adrian.boicean@ulbsibiu.ro; 6 Department of Pediatrics III, George Emil Palade University of Medicine, Pharmacy, Science and Technology of Targu Mures, Gheorghe Marinescu Street No. 38, 540136 Targu Mures, Romania; maria-oana.marginean@umfst.ro

**Keywords:** depression, anxiety, ulcerative colitis, irritable bowel disease with diarrhea

## Abstract

**Background**: Common mental disorders are an underdiagnosed comorbidity, which can significantly worsen the prognosis of the main disease and decrease the quality of life. We aimed to investigate the prevalence of depression and anxiety in a cohort of irritable bowel syndrome with diarrhea (IBS-D) and ulcerative colitis (UC) patients and to evaluate the risk factors for their occurrence. **Materials and Methods:** A total of 112 patients were evaluated. Multivariable analysis was used to determine associations between patient factors and common mental disorders, evaluated with PHQ-9 and GAD-7 questionnaires. **Results:** We found a significantly higher prevalence of moderate and severe anxiety among patients with IBS-D, when compared with the UC group (*p* < 0.01). Linear regression analysis revealed an inverse association between anti-TNF-alpha monoclonal antibodies treatment and a higher PHQ-9 score (*p* = 0.02). Multivariate analysis revealed that, in patients with UC, the presence of children has been associated with a higher GAD-7 score (*p* = 0.01), both individually and in combination with a higher duration of the disease. (*p* < 0.01). For IBS-D, a combination of active employment status and religious belief, active employment status and higher educational level, as well as religious belief and the presence of children correlated with higher GAD-7 scores (*p* = 0.03, *p* = 0.03 and *p* = 0.02, respectively). **Conclusions:** Infliximab used in the treatment for UC improved the parameters of depression. Patients with UC who have university education and a longer duration of the disease are at increased risk of developing depression and anxiety, especially if they have children in care. Regarding IBS-D patients who have an active work status, religious beliefs and caregivers are at increased risk of developing anxiety.

## 1. Introduction

Ulcerative colitis (UC) and irritable bowel syndrome with diarrhea (IBS-D) are chronic diseases of the gastrointestinal tract that exhibit approximately the same symptoms, but have different etiopathogenetic substrate [1]. UC is an inflammatory bowel disease (IBD) that affects the colon mucosa and most frequently involves remissions and flares-ups [2]. Irritable bowel syndrome (IBS) is a chronic, recurrent functional gastrointestinal condition characterized by abdominal pain, associated with intestinal transit disorders, in the absence of organic lesions. IBS is classified into subtypes based on the dominant model [3]. IBS-D is estimated to account for approximately one third of IBS cases [3,4].

Mental health is a very important but overlooked aspect in patients with chronic diseases. The most common mental disorders (mCMD) in patients with UC or IBS-D are anxiety and depression [5]. In general, anxiety coexists with depression [6]. Beginning in the 1930s, studies showed that emotional factors and personal experiences were correlated with the severity of IBD [7]. One recent study showed that patients with IBD and mental health disease generated significantly higher costs to the health system compared with patients without mental disorders [8]. Moreover, mental health disorders decreased quality of life in IBD patients [9] and led to poor treatment compliance [10]. Fairbrass et al., through a large study that included 718 patients with IBD followed-up for 6.5 years, showed that anxiety and depression are risk factors for a poor prognosis [11].

Recently published studies have suggested that depression may be related to the direct effect of inflammation on the brain [12,13,14], with systemic proinflammatory cytokines being the culprit. One of these cytokines, tumor necrosis factor-α (TNF-α), has also been implicated in UC pathogenesis [15,16]. The relationship between mCMD and disorders such as IBD and IBS-D seems to be bidirectional. On the one hand, patients who initially do not present mCMD could develop symptoms of depression or anxiety in the future [17], suggesting the involvement of the brain–gut axis. On the other hand, existing mCMD can lead to the exacerbation of UC or IBS-D symptoms [11,18,19,20]. In a study conducted by Jordi et al., it became clear that there is a genetic link between depression and IBD [21]. Thus, the alleles of two simple nucleotides polymorphisms (rs588765-TC, rs2522833 C allele) were negatively associated with depressive symptoms among IBD patients, and very importantly, a combination of alleles (rs588765-TC) was highlighted in patients with low IBD activity [21].

The prevalence of depression varies between 21 and 25% [22], or 7 and 59% [6], whereas that of anxiety between 19.1 and 35% [21], and 15.5 and 40% [6]. Although the prevalence of these common mood disorders is high, the literature data suggest that anxiety and/or depression are not risk factors for the onset of IBD [18,20], but might be related to their flare-ups [6]. Several factors may contribute to the risk of depression and anxiety in patients with chronic colonic diseases, characterized by abdominal pain and diarrhea. On the one hand, psychological dysfunction may be secondary to activity, form and/or side effects of treatment (for example, corticosteroids) [18,23]. On the other hand, social deprivation [17], poor socioeconomic status, previous surgery [18], older age [18,24], and female sex [17,25] pose a high risk of the development of depression or anxiety.

In the case of IBS, between 50% and 90% of patients present mCMD [4,26]. Moreover, in the literature, some studies highlight the association between mCMD and IBS-D [27,28], while others do not report the same correlation [29,30]. This gap in the research implies additional efforts to find out the magnitude of the problem. Timely diagnosis, psychological counselling and prompt treatment of depression or anxiety can enhance long-term results.

Therefore, the aim of this study was to comparatively assess the prevalence of depression and anxiety in subjects with UC and IBS-D from a tertiary center, through the help of standardized psychological tests, and to evaluate the risk factors for their occurrence.

## 2. Materials and Methods

An observational, non-interventional study was conducted to evaluate depressive and anxious symptoms in order to evaluate the interrelationships between these variables and the characteristics of a Romanian cohort of patients with UC and IBS-D. We evaluated patients from the Department of Gastroenterology, Mures County Clinical Hospital, Romania, between March 2022 and March 2023, using the PHQ-9 and GAD-7 questionnaires. These represent reliable screening tools for depression and anxiety (Cronbach’s alpha 0.86 and 0.91, respectively) [31]. The inclusion criteria were as follows: a diagnosis of UC or IBS-D; at least 6 months from diagnosis; the absence of other severe chronic illnesses. The exclusion criteria were as follows: patients with cognitive function impairment; corticosteroid therapy; the presence of other severe systemic illnesses (neoplasia, orthopedic, cardiovascular, or respiratory pathologies); and patients on sedatives, antidepressants or antianxiety medication at enrolment.

Generalized Anxiety Disorder-7 (GAD-7) is a 7-item self-report questionnaire assessing levels of anxiety, which has demonstrated promise as a scale with good clinical applicability and powerful psychometric characteristics [32], and has demonstrated good reliability and structural validity in several studies, with a sensitivity of 89% and specificity of 82% [33,34]. Finally, scores of ≥ 5, 10 and 15, respectively, represent cut-off values for mild, moderate and severe anxiety [32]. In addition, depression was assessed using Patients Health Questionnaire 9 (PHQ-9). Scores of ≥5, 10, and 15 points on the PHQ-9 are indicative of mild, moderate, and severe depression, respectively [33]. PHQ-9 and GAD-7 tests, translated into Romanian, were completed by all study participants. Patients were given instructions and then completed all questionnaires independently after signing an informed consent form. We did not need special approvals for using tests to quantify depression (PHQ-9) and anxiety (GAD-7).

The diagnosis of UC was made according to the criteria Organizations of Crohn’s and Colitis guidelines [35], whereas the Montreal classification was used for the classification of colonic disease extension [36]. The Ulcerative Colitis Disease Activity Index (UCDAI) was used to classify disease severity into mild (< 3(, moderate (between 3 and 7) and severe (> 7), through a composite assessment of clinical symptoms: stool frequency, rectal bleeding and endoscopic severity [37]. IBS-D was diagnosed using Rome IV criteria [38]. Colonoscopies were performed under Propofol sedation, with careful monitoring by an anesthesiologist. Prior to colonoscopy, patients underwent an examination for ovum and parasites in the stool and a fecal culture test, including mycological and Clostridium Difficile toxin tests with negative results.

The following demographic data were collected: age, gender, religion, marital status, and children. Educational levels were stratified depending on the latest finalized educational degree into elementary school, high school and university degree. Employment status was classified as employed, without occupation (unemployed) and retired. The disease (UC and IBS-D)-derived parameters that we focused on for their impact on the patients’ mental health included disease duration (time from the disease’s onset in months), extension, severity and therapeutic line.

### 2.1. Ethics Statement

This study was approved by the Ethics Committees of the County Clinical Hospital of Târgu Mureș (Institutional review board no. 4872/24.05.2022), and that of the George Emil Palade University of Medicine, Pharmacy, Science and Technology of Târgu Mureș (Institutional review board no 1804/22.06.2022), and it was performed following the principles stated in the Declaration of Helsinki. All subjects signed an informed consent form before they were enrolled in the study.

### 2.2. Statistical Analysis

Statistical analysis was performed with the help of GraphPad software, version 10.3.1. A confidence interval of 95% was applied to the test conducted, corresponding to a statistical significance threshold of *p* = 0.05. Descriptive statistics analysis was conducted, with data being expressed as mean ± standard deviation (SD) or as a percentage. Kolmogorov–Smirnov was the normality test chosen to assess the Gaussian/non-Gaussian distribution of the analyzed data. Mean comparison was performed with the help of the t Student test or Mann–Whitney test, depending on the data distribution. Binary data analysis was conducted using the Chi-square test. To investigate parameter association, the non-parametric Spearman correlation test was used. In the case of association trends between binary variables and quantitative variables, linear regression analysis was applied. Multivariate analysis was applied to investigate how disease severity parameters, lifestyle and socio-economic cofounders influence Gad-7 and PHQ-9 score, taken individually or as part of the combined model of 2 variables.

## 3. Results

The study sample consisted of a total number of 112 patients, divided into two groups: group 1—diagnosed with UC, and group 2—diagnosed with IBS-D.

Table 1 depicts a comparison of social-derived parameters between group 1 and group 2, as well as of the two scores and their corresponding classifications between the same groups. A significantly younger mean age was found in group 1 (42.91 ± 15.48 versus 49.91 ±17.90, *p* = 0.02), as well as a compellingly higher prevalence of male sex in the same group, when compared with group 2, in which the majority of patients belonged to the female sex (*p* < 0.01, OR = 4.25). No significant discrepancies were found in terms of rural/urban background, religion, occupation or the presence of children in the family, nor of the time that passed since the moment of diagnosis between the two groups. When comparing level of education, a higher level of studies was found among these patients with UC (*p* < 0.01). In terms of the questionnaire-derived scores, PHQ-9 scores were similar between the two groups, but GAD-7 scores were significantly higher among patients with IBS-D (11.00 ± 3.82 versus 6.15 ± 3.66, *p* < 0.01). Moreover, these patients were more frequently classified with moderate or severe anxiety than those with UC (*p* < 0.01).

Non-parametric Pearson correlation was employed to assess correlations between UC parameters of severity, timeline from disease diagnosis and depression/anxiety severity scores. However, no significant results were found (Table 2).

In similar fashion, for patients belonging to group 2, time from diagnosis was correlated with PHQ-9, GAD-7 scores and their classification, without any significant results (Table 3).

**Figure 1:**
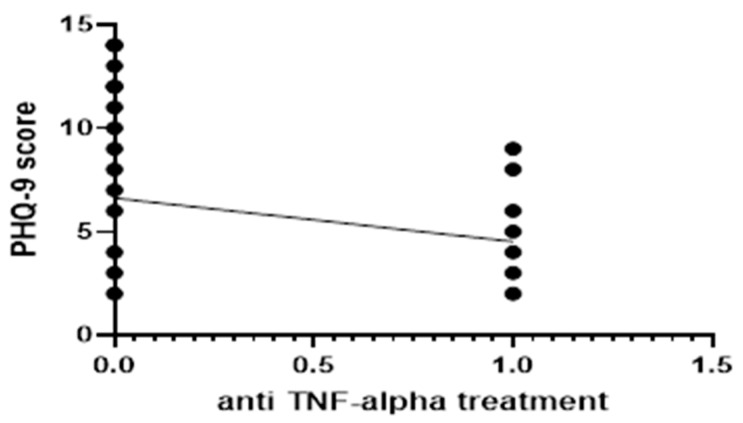
Linear regression analysis for correlation between anti TNF-alpha treatment and PHQ-9 score in group 1.

For group 1, correlations between patients on immunosuppressive treatment (Azathioprine) with PHQ-9 and GAD-7 scores were found, but without significant results; *p* = 0.4516 and *p* = 0.4397, respectively. In particular, an association between the presence of anti-TNF-alpha therapy and both scores was investigated in patients with UC. Linear regression analysis revealed an inverse association between periodic anti-TNF-alpha monoclonal antibodies treatment and a higher PHQ-9 score (*p* = 0.02, as seen in Figure 1). The presence of biological therapy did not seem, however, to correlate with GAD-7 score (*p* = 0.95).

Multivariate analysis was performed to assess the impact of cofounders over the GAD-7 anxiety score in patients with UC. Individual parameters of severity, lifestyle, social and educational factors were assessed individually, as well as in combination of two parameters (Table 4). The presence of children has been associated with a higher GAD-7 score (*p* = 0.01), both individually and in combination with a higher duration of disease understanding after diagnosis (*p* < 0.01). Furthermore, a higher level of studies and a prolonged disease duration seem also to negatively impact GAD-7 scores (*p* < 0.01). No other cofounders significantly impacted the value of GAD-7 scores, either individually or in combination.

In Table 5, the same type of statistical analysis was employed to assess how the same confounders impact the PHQ-9 scores in patients belonging to group 1. Individual parameters were not significantly associated with depression severity. A unique significant positive association was seen when taking into consideration both study level and time from diagnosis (*p* < 0.01). No other significant associations were found for parameter pairings.

As in the case of group 1, multivariate analysis was performed to assess social- and lifestyle-derived cofounder impact on GAD-7 score in patients belonging to group 2 diagnosed with IBS-D (Table 6). Although no individual associations were found, a combination of active employment status and religious belief, active employment status and higher educational level, as well as religious belief and the presence of children correlated with higher GAD-7 scores (*p* = 0.03, *p* = 0.03 and *p* = 0.02, respectively).

In the case of the PHQ-9 depression score, none of the individual/combined confounders seemed to influence its values in patients with IBS-D, as depicted in Table 7.

## 4. Discussion

This study found that the prevalence of depression and anxiety was 54% and 59%, and 64% and 90%, respectively, among UC and IBS-D patients. For UC patients, the literature data prevalence is similar to our results, as Sneineh et al. highlighted a prevalence of depression of 58.6% and anxiety of 65.7% [39]. Contradictorily, the results of two studies from Iran conducted only on UC patients found a depression prevalence of 29.2% and anxiety of 40%, as well as 43.5% and 81.5% [40,41]. In case of IBS-D, the literature highlights results that are similar to ours [4], or with significant variation: Banerjee et al., in a study that included 50 patients, found anxiety and depression in 37.1% and 44% patients [26]. We consider these differences to be likely caused by varying perceptions of each patient, using a different cut-off level, and by the lack of a disease-specific tool. Different questionnaires were used to assess depression and anxiety, such as the Hospital Anxiety and Depression Scale [6,21], Hamilton Depression or Anxiety Scale [26,42] or the depression, anxiety and stress scale 21 [4]. Moreover, the degree of depression and anxiety could be overestimated due to self-assessment and due to the fact that certain questions that are included in the depression assessment questionnaire, such as those related to appetite or energy, may overlap with symptoms characteristic of UC or IBS-D.

Our data show a significantly higher prevalence of moderate and severe anxiety among patients with IBS-D, as suggested by the value of the GAD-7 scores, when comparing the two study groups. As a matter of fact, IBS-D has been linked with depression, with some authors tracing back adverse childhood events in relation to symptom severity [43,44]. Depression scores such as PHQ_9 have been positively linked to symptom severity of IBS [44]. This further translates into a need for symptom control through depression management. Tricyclic antidepressants in low dosages, such as amitriptyline, have been proposed through randomized controlled trials as a second line therapy for patients with IBS [45]. For the evaluation of symptom burden in patients with IBS, an experience sampling method-based patient-reported outcome measure (ESM-PROM) has been shown to be more accurate for the assessment of real-time gastrointestinal symptom burden. Both GAD-7 and PHQ-9 have correlated moderately with ESM-PROM [46]. In the literature, we did not find any studies comparing mCMD between patients with UC or IBS-D, only in terms of quality of life.

Many studies have reported the existence of a correlation between mCMD and the disease activity [21,39,40,47], as well as the recurrence of symptoms [6,18,21]. Our study does not highlight correlations between PHQ-9/GAD-7 scores and severity parameters or timeline from diagnosis. Stroie et al. did not obtain correlations between mCMD and timeline from diagnosis [48], and Askar et al. did not detect significant associations between mCMD and UC, even if other questionnaires were used and other cut-offs were established [42]. The most relevant explanations could be the fact that the studies included a smaller number of patients and/or the inclusion criteria were different.

Instead, the multivariate analysis highlights the fact that in patients with UC, the presence of anxiety correlates significantly with a longer duration since diagnosis, university studies and dependent children. Likewise, the long duration of the disease in patients with university education led to an increased risk of developing depression. Similar to our findings, several studies have correlated the occurrence of depression and anxiety with the presence of caregivers. More specifically, married participants with children, probably because of the greater number of responsibilities that makes them feel overwhelmed, may experience mCMD [49]. In other studies, unemployment [50], elderly patients and patients who are separated, divorced or widowed [24] have been described as at risk of the occurrence of mCMD.

Regarding IBS-D, significantly impacting social and lifestyle cofounders were identified only in the case of anxiety. The multivariate analysis performed showed that a combination of active employment status and religious belief, active employment status and higher educational level, as well as religious belief and the presence of children correlated with higher GAD-7 scores (*p* = 0.03, *p* = 0.03 and *p* = 0.02, respectively). These findings might be related to other literature data results. Firstly, religious belief and integration in a spiritual community seems to represent a coping mechanism for chronic disorders. As a matter of fact, one study showed that both positive and negative religious coping have been significantly related to depression in patients with IBS, independently of the type of religious practice, with Roman catholic taken as religion reference [51]. A recent study carried out on 100 patients obtained contradictory results to ours; more specifically, unemployed patients were found to have a higher risk of anxiety [4]. However, our results regarding the positive relationship between university education and the presence of mCMD are consistent with those found in the previous study, as well as in another report by Khanna et al. In both studies, a higher percentage of subjects diagnosed with IBS had achieved higher educational degrees [4,52]. As stress is a well-known trigger for IBS, it is unsurprising that subjects who pursue university studies also present mCMD more frequently, including depression, as they are often more exposed to pressure in the academic environment, due to workload and the will to climb up the career ladder, which can also attract familial relationship malfunction.

In our study, UC patients with anti TNF-alpha monoclonal antibody therapy presented significantly lower PHQ-9 scores. These results are in accordance with previous reports that sustain that anti-TNF-alpha treatment improves short and long-term depression-associated symptoms in patients with IBD [53]. Another longitudinal study observed significant improvement in depression and anxiety scores in patients undergoing immunomodulatory and/or TNF-alpha therapy [54]. A study by Chen et al. conducted on rats reported that if UC-like inflammation is induced, it will cause anxiety- and depression-like behaviors, as well as continuous abdominal discomfort. Symptoms may persist even after the inflammatory response has subsided, suggesting that inflammation may induce long-lasting changes in the expression of neurotrophins and ion channels in afferent neurons through epigenetic programming [14], which further implies that different mediators could be involved. While it is yet unclear how these mediators act on the brain, it appears that anti-TNF-alpha therapy can act on the gut–brain axis, by hindering mucosal inflammation and improving microbiome imbalances that come with active UC [53,55]. Another study published in 2020 revealed that the serum TNF-a levels were higher in patients with severe depression than in healthy controls. Following the antidepressant treatment, the TNF-alpha levels were significantly decreased and comparable to those of healthy controls [56]. However, a cohort study that analyzed data from the French national hospital discharge abstract database (PMSI) between 2008 and 2014 showed that psychotic episodes have occurred in patients treated for UC (HR = 5.43 [2.01; 14.6]) [57].

Early recognition of depression and anxiety mood disorders is crucial to ensure adherence to anti-TNF-alpha therapy in patients with IBD who might require it in the long term for disease management, according to Dolovich et al. [58]. Understanding the pathophysiological changes associated with UC and neuroinflammation may help develop more appropriate pharmaceutical targeted treatment for depression associated with UC.

In this study, both moderately severe symptoms of depression and those of anxiety were more severe in patients with IBS than in patients with UC. The same associations are described by Geng and collaborators in a meta-analysis that included 22 studies with a total of 2292 subjects (1244 IBS and 1048 IBD patients) [59]. Although the symptomatology of these conditions is similar [1], they are associated with mood disorders in different ways, which could be due to several reasons. On the one hand, the use of self-assessment tests introduces a degree of subjectivity to these evaluations. On the other hand, IBS is a functional disease that is considered to be strongly influenced by the psychological traits of the individual, while UC is an organic disease in which mood disorders are often secondary to the condition. Additionally, IBS-D patients have higher levels of psychological distress and less support for their physical complaints compared to UC patients, due to the lack of organic lesions but with dysfunction in the brain–gut pathways [59].

Like every study, our study has some limitations. Firstly, the data are self-reported and cannot be independently verified. Secondly, the psychological state of the patients was evaluated at the time of the consultation using questionnaires, not specific analyses. Thirdly, the relatively small number of patients included in study (patients with comorbidities, with corticosteroid treatment that could contribute to the development of psychiatric disorders were excluded from the study) is another setback of our research. Despite these limitations, our study shows that the current approach to mental health in this vulnerable population with chronic intestinal symptoms is not appropriate. The presence of anxiety and depression must be assessed at the initial consultation, and then periodically, followed by psychological intervention when necessary. This could lead to improved outcomes in UC or IBS-D patients. The seriousness of the problem also emerges from the results highlighted by a Cochrane review, which showed that in the adult population with mCMD and IBD, the performance of psychotherapy reflects on the quality of life [60]. In the future, research is needed to help standardize diagnostic questionnaires suitable for mCMD with questions suitable for UC and IBS-D, such as The Luebeck Interview for Psychosocial Screening in Patients with IBD [61] or the World Health Organization’s Composite International Diagnostic Interview [62]. Moreover, it is necessary to standardize the approach after the quantification and diagnosis of anxiety and depression.

## 5. Conclusions

Patients with IBS-D are more likely to have anxiety and moderate or severe depression compared to patients with UC. In particular, those subjects with IBS-D who have an active work status, have religious beliefs and are caregivers are at increased risk of developing anxiety. Patients with UC who have university education, children in care and a higher duration of the disease are at increased risk of developing anxiety. Moreover, those individuals with university education and higher duration of disease are at increased risk of developing depression. Infliximab used in the treatment for UC improved the parameters of depression.

The increased prevalence of these mental disorders calls for mental screening of each person diagnosed with UC or IBS-D at the initial consultation and follow-up. The identification, using simple diagnostic tools, of anxiety and depression in these patients, can be important in improving treatment adherence and increasing quality of life.

## Figures and Tables

**Table 1:** Comparison of characteristics and depression severity scores between groups 1 and 2.

Parameter		Group 1 (*n* = 58) (Mean ± SD/% *)	Group 2 (*n* = 54) (Mean ± SD/% *)	*p* Value
Age		42.91 ± 15.48	49.91 ±17.90	0.02
Gender	Female	19.64	34.82	<0.0001; OR = 4.25
	Male	32.14	13.39	
Background	Urban	36.60	26.78	0.83; OR = 1.12
	Rural	15.17	12.5	
Occupation	Retired	8.92	14.28	0.05
	Employed	33.92	25	
	Unemployed	8.92	13.39	
Children	Yes	32.14	34.82	0.25, OR = 0.62
	No	19.64	13.39	
Religious belief	Yes	27.67	31.25	0.22, OR = 0.62
	Atheist	24.10	16.96	
Studies	Primary school	12.5	24.10	<0.0001
	High school	19.64	16.07	
	University	19.64	0.08	
Time from diagnosis (months)		22.34 ± 15.14	20.43 ± 12.09	0.72
PHQ-9 score classification	None to minimal depression	24.10	16.96	0.17
	Mild depression	21.42	17.85	
	Moderate depression	6.25	12.5	
	Moderately severe depression	0	0.8	
GAD-7 score classification	Normal to mild anxiety	22.32	4.46	<0.0001
	Mild anxiety	24.10	13.39	
	Moderate anxiety	4.46	26.78	
	Severe anxiety	0.8	3.57	
PHQ-9 score (absolute value)		5.86 ± 3.35	7.55 ± 4.80	0.12
GAD-7 score (absolute value)		6.15 ± 3.66	11.00 ± 3.82	<0.0001

Legend: *—data expressed as mean ± SD for numerical variables and % for categorical variables; OR—odds ratio; SD—standard deviation.

**Table 2:** Correlation between UC parameters of severity, time from disease diagnosis and depression severity scores.

Parameter	Correlated Parameter	*p* Value, r Coefficient
Time from diagnosis (months)	PHQ−9 score (absolute value)	*p* = 0.30, r = −0.13 (CI: −0.39–0.13)
Disease severity ^a^	PHQ−9 score (absolute value)	*p* = 0.92, r = −0.01 (CI: −0.27–0.24)
UCDAI score	PHQ−9 score (absolute value)	*p* = 0.69, r = −0.07 (CI: −0.39–0.27)
Time from diagnosis (months)	GAD−7 score (absolute value)	*p* = 0.89, r = −0.02 (CI: −0.28–0.24)
Disease severity ^a^	GAD−7 score (absolute value)	*p* = 0.85, r = −0.02 (CI: −0.28–0.24)
UCDAI score	GAD−7 score (absolute value)	*p* = 0.34, r = 0.16 (CI: −0.18–0.47)
Time from diagnosis (months)	PHQ−9 score (classification ^b^)	*p* = 0.11, r = −0.21 (CI: −0.45–0.05)
Disease severity ^a^	PHQ−9 score (classification ^b^)	*p* = 0.36, r = 0.12 (CI: −0.15–0.37)
UCDAI score	PHQ−9 score (classification ^b^)	*p* = 0.83, r = 0.03 (CI: −0.37–0.30)
Time from diagnosis (months)	GAD−7 score (classification ^c^)	*p* = 0.95, r = 0.007 (CI: −0.25–0.27)
Disease severity ^a^	GAD−7 score (classification ^c^)	*p* = 0.77, r = −0.03 (CI: −0.29–0.22)
UCDAI score	GAD−7 score (classification ^c^)	*p* = 0.27, r = 0.19 (CI: −0.16–0.50)

Legend: a—as assessed in accordance with the UCDAI score. b—score 0–4, none to minimal depression; score 5–9, mild depression; score 10–14, moderate depression; score 15–19, moderately severe depression; score 20–27, severe depression. c—score 0–4, normal to mild anxiety; score 5–9, mild anxiety; score 10–14, moderate anxiety; score 15–21, severe anxiety. CI—confidence interval. UCDAI—ulcerative colitis disease activity index.

**Table 3:** Correlation between time from disease diagnosis for IBS-D patients and depression/anxiety severity scores.

Time from diagnosis (months)	PHQ−9 score (absolute value) ^a^	*p* = 0.46, r = 0.09 (CI: −0.17–0.36)
Time from diagnosis (months)	PHQ−9 score (classification) ^a^	*p* = 0.88, r = 0.01 (CI: −0.25–0.29)
Time from diagnosis (months)	GAD−7 score (absolute value) ^b^	*p* = 0.70, r = −0.05 (CI: −0.32–0.22)
Time from diagnosis (months)	GAD−7 score (classification) ^b^	*p* = 0.44, r = −0.10 (CI: −0.37–0.17)

Legend: a—score 0–4, none to minimal depression; score 5–9, mild depression; score 10–14, moderate depression; score 15–19, moderately severe depression; score 20–27, severe depression. b—score 0–4, normal to mild anxiety; score 5–9, mild anxiety; score 10–14, moderate anxiety; score 15–21, severe anxiety. CI—confidence interval.

**Table 4:** Multiple linear regression for the association between severity parameters, lifestyle and educational cofounders and GAD-7 score in patients with UC.

Variable	Estimate	Standard Error	95% CI	t Value	*p* Value
Intercept	19.21	15.46	−13.15–51.56	1.24	0.23
Gender (ref. = male sex)	−13.17	13.61	−41.65–15.31	0.96	0.34
Background (ref. = rural background)	−2.38	9.70	−22.68–17.91	0.24	0.80
Children (ref.= no children)	−24.45	8.74	−42.76–−6.15	2.79	0.01
Severity (ref.= mild form)	−1.73	5.88	−14.04–10.57	0.29	0.77
Colonic disease extension	−3.22	6.69	−17.23–10.79	0.48	0.63
Religion (ref.= atheist)	−7.56	8.17	−24.67–9.54	0.92	0.36
Studies (ref.= primary school)	0.11	4.48	−9.26–9.49	0.02	0.98
Time from diagnosis (months)	0.64	0.35	−0.08–1.38	1.85	0.08
Gender + background	−6.01	3.59	−13.53–1.49	1.67	0.11
Gender + children	1.59	3.62	−6.00–9.18	0.44	0.66
Gender + severity	4.81	2.83	−1.11–10.73	1.70	0.10
gender + colonic disease extension	4.47	2.61	−0.99–9.94	1.71	0.10
gender + religion	3.00	3.86	−5.09–11.10	0.77	0.44
Gender + studies	0.91	1.80	−2.86–4.68	0.50	0.62
Gender + time from diagnosis	−0.10	0.12	−0.36–0.15	0.82	0.42
Background + children	2.17	3.67	−5.52–9.86	0.59	0.56
Background + severity	−1.16	2.60	−6.60–4.27	0.45	0.65
Background + colonic disease extension	−4.50	2.77	−10.32–1.30	1.62	0.12
Background + religion	4.97	3.69	−2.76–12.71	1.34	0.19
Background + studies	2.29	1.93	−1.74–6.33	1.19	0.24
Background + UC duration	0.21	0.14	−0.08–0.50	1.51	0.14
Children + severity	3.19	2.55	−2.14–8.53	1.25	0.22
Children + colonic disease extension	−0.15	1.66	−3.63–3.32	0.09	0.92
Children + religion	4.30	3.72	−3.50–12.11	1.15	0.26
Children + studies	2.36	1.86	−1.54–6.27	1.26	0.22
Children + time from diagnosis	0.48	0.11	0.24–0.73	4.12	<0.0001
Severity + colonic disease extension	1.71	1.48	−1.39–4.82	1.15	0.26
Severity + religion	0.25	3.10	−6.24–6.75	0.08	0.93
Severity + studies	−1.55	1.49	−4.69–1.58	1.03	0.31
Severity + time from diagnosis	−0.04	0.07	−0.19–0.11	0.53	0.60
Colonic disease extension + religion	2.91	2.57	−2.47–8.30	1.13	0.27
Colonic disease extension + studies	2.12	1.82	−1.70–5.94	1.16	0.26
Colonic disease extension + time from diagnosis	−0.14	0.07	−0.30–0.02	1.81	0.08
Religion + studies	−2.00	1.94	−6.08–2.07	1.02	0.31
Religion + time from diagnosis	−0.25	0.17	−0.62–0.10	1.47	0.15
Studies + time from diagnosis	−0.30	0.10	−0.51–0.09	2.99	<0.0001

Legend: CI—confidence interval.

**Table 5:** Multiple linear regression for the association between severity parameters, lifestyle and educational cofounders and PHQ-9 score in patients with UC.

Variable	Estimate	Standard Error	95% CI	t Value	*p* Value
Intercept	39.57	16.58	4.86–74.27	2.38	0.02
Gender (ref. = male sex)	15.73	14.60	−14.82–46.28	1.07	0.29
Background (ref. = rural background)	−10.48	10.40	−32.25–11.29	1.00	0.32
Children (ref.= no children)	10.40	9.37	−9.23–30.03	1.10	0.28
Severity (ref.= mild form)	−8.08	6.30	−21.28–5.11	1.28	0.21
Colonic disease extension	−13.74	7.18	−28.77–1.28	1.91	0.07
Religion (ref.= atheist)	4.39	8.76	−13.96–22.74	0.50	0.62
Studies (ref.= primary school)	−7.45	4.80	−17.51–−2.61	1.55	0.13
Time from diagnosis (months)	−0.31	0.37	−1.09–0.47	0.83	0.41
Gender + background	−3.09	3.85	−11.15–4.96	0.80	0.43
Gender + children	3.78	3.89	−4.36–11.93	0.97	0.34
Gender + severity	−2.06	3.03	−8.41–4.29	0.68	0.50
gender + colonic disease extension	1.80	2.80	−4.06–7.66	0.64	0.52
gender + religion	−8.81	4.14	−17.50–−0.13	2.12	0.04
Gender + studies	−2.24	1.93	−6.29–1.80	1.16	0.26
Gender + time from diagnosis	−0.16	0.13	−0.44–0.11	1.21	0.24
Background + children	0.56	3.94	−7.68–8.81	0.14	0.88
Background + severity	1.28	2.78	−4.55–7.11	0.46	0.65
Background + colonic disease extension	3.03	2.97	−3.20–9.26	1.01	0.32
Background + religion	1.29	3.96	−6.99–9.59	0.32	0.74
Background + studies	1.49	2.07	−2.83–5.83	0.72	0.47
Background + UC duration	−0.13	0.15	−0.45–0.17	0.91	0.36
Children + severity	0.74	2.73	−4.98–6.48	0.27	0.78
Children + colonic disease extension	0.11	1.78	−3.62–3.84	0.06	0.95
Children + religion	−3.35	4.00	−11.72–5.02	0.83	0.41
Children + studies	−4.41	2.00	−8.60–−0.22	2.20	0.04
Children + time from diagnosis	−0.10	0.12	−0.37–0.15	0.85	0.40
Severity + colonic disease extension	−0.59	1.59	−3.92–2.73	0.37	0.71
Severity + religion	0.71	3.33	−6.26–7.68	0.21	0.83
Severity + studies	1.66	1.60	−1.69–5.02	1.03	0.31
Severity + time from diagnosis	0.23	0.08	0.06–0.40	2.90	<0.0001
Colonic disease extension + religion	−0.93	2.76	−6.70–4.84	0.33	0.74
Colonic disease extension + studies	3.48	1.96	−0.61–7.58	1.78	0.09
Colonic disease extension + time from diagnosis	0.13	0.08	−0.04–0.31	1.61	0.12
Religion + studies	1.64	2.09	−2.73–6.01	0.78	0.44
Religion + time from diagnosis	−0.07	0.18	−0.47–0.31	0.41	0.68
Studies + time from diagnosis	−0.08	0.10	−0.31–0.14	0.78	0.44

Legend: CI—confidence interval.

**Table 6:** Multiple linear regression for the association between lifestyle and social cofounders and GAD-7 score in patients with IBS-D.

Variable	Estimate	Standard Error	95% CI	t Value	*p* Value
Intercept	12.17	6.96	−2.16–26.50	1.74	0.09
Gender (ref. = female sex)	−7.72	8.29	−24.81–9.36	0.93	0.36
Background (ref. = rural background)	−4.35	5.83	−16.36–7.66	0.74	0.46
Occupation (ref.= no occupation)	6.32	3.71	−1.32–13.98	1.70	0.10
Children (ref.= no children)	−1.98	11.74	−26.16–22.19	0.16	0.86
Religion (ref.= atheist)	−4.65	7.76	−20.63–11.33	0.60	0.55
Studies (ref.= primary school)	0.06	5.01	−10.26–10.40	0.01	0.99
Time from diagnosis (months)	−0.21	0.25	−0.73–0.30	0.84	0.40
Gender + background	2.40	3.97	−5.77–10.59	0.60	0.55
Gender + occupation	0.52	2.70	−5.04–6.08	0.19	0.84
Gender + children	−0.35	5.38	−11.45–10.73	0.06	0.94
Gender + religion	−3.94	5.22	−14.70–6.82	0.75	0.45
Gender + studies	3.27	3.34	−3.61–10.17	0.98	0.33
Gender + time from diagnosis	0.06	0.23	−0.41–0.53	0.27	0.78
Background + occupation	−2.23	2.21	−6.79–2.32	1.00	0.32
Background + children	−8.64	4.54	−18.01–0.71	1.90	0.06
Background + religion	−0.27	5.00	−10.58–10.03	0.05	0.95
Background + studies	3.39	3.01	−2.81–9.60	1.12	0.27
Background + time from diagnosis	0.16	0.23	−0.31–0.64	0.71	0.47
Occupation + children	2.77	3.61	−4.67–10.21	0.76	0.45
Occupation + religion	6.72	2.93	0.68–12.76	2.29	0.03
Occupation + studies	−4.62	2.00	−8.75–−0.49	2.30	0.03
Occupation + time from diagnosis	−0.02	0.10	−0.24–0.18	0.27	0.78
Children + religion	11.09	4.64	1.51–20.66	2.38	0.02
Children + studies	−1.09	3.53	−8.36–6.18	0.30	0.76
Children + time from diagnosis	0.002	0.18	−0.37–0.37	0.01	0.98
Religion + studies	2.40	3.42	−4.65–9.45	0.70	0.49
Religion + time from diagnosis	−0.59	0.45	−1.52–0.34	1.30	0.20
Studies + time from diagnosis	0.15	0.22	−0.32–0.62	0.65	0.51

Legend: CI—confidence interval.

**Table 7:** Multiple linear regression for the association between lifestyle and social cofounders and PHQ-9 score in patients with IBS-D.

Variable	Estimate	Standard Error	95% CI	t Value	*p* Value
Intercept	5.80	8.24	−11.18–22.79	0.70	0.48
Gender (ref. = female sex)	−6.06	9.83	−26.31–14.18	0.61	0.54
Background (ref. = rural background)	−0.38	6.91	−14.62–13.85	0.05	0.95
Occupation (ref.= no occupation)	4.16	4.40	−4.90–13.23	0.94	0.35
Children (ref.= no children)	−9.30	13.91	−37.96–19.34	0.67	0.51
Religion (ref.= atheist)	−3.21	9.19	−22.15–15.72	0.35	0.73
Studies (ref.= primary school)	1.83	5.94	−10.41–14.08	0.31	0.76
Time from diagnosis (months)	0.13	0.30	−0.48–0.75	0.46	0.65
Gender + background	−6.15	4.71	−15.86–3.54	1.30	0.20
Gender: occupation	−0.03	3.20	−6.63–6.56	0.01	0.99
Gender + children	−7.01	6.38	−20.16–6.13	1.10	0.28
Gender + religion	6.01	6.19	−6.74–18.77	0.97	0.34
Gender + studies	−2.92	3.964	−11.08–5.24	0.73	0.46
Gender + time from diagnosis	0.56	0.27	−0.0003–1.12	2.05	0.05
Background + occupation	2.33	2.62	−3.06–7.73	0.89	0.38
Background + children	0.73	5.38	−10.36–11.83	0.13	0.89
Background + religion	−1.54	5.93	−13.76–10.67	0.26	0.79
Background + studies	1.34	3.57	−6.01–8.696	0.37	0.71
Background + time from diagnosis	−0.1749	0.2748	−0.74–0.39	0.63	0.53
Occupation + children	−1.74	4.28	−10.56–7.07	0.40	0.68
Occupation + religion	0.56	3.47	−6.59–7.71	0.16	0.87
Occupation + studies	−1.59	2.37	−6.48–3.30	0.67	0.50
Occupation + time from diagnosis	−0.10	0.12	−0.36–0.14	0.87	0.39
Children + religion	3.89	5.50	−7.45–15.23	0.70	0.48
Children + studies	0.24	4.18	−8.36–8.86	0.06	0.95
Children + time from diagnosis	0.30	0.21	−0.13–0.75	1.43	0.16
Religion + studies	0.11	4.05	−8.24–8.46	0.02	0.97
Religion + time from diagnosis	−0.002	0.53	−1.10–1.10	0.004	0.99
Studies + time from diagnosis	−0.06	0.27	−0.62–0.49	0.24	0.80

Legend: CI—confidence interval.

## Data Availability

The raw data supporting the conclusions of this article will be made available by the authors on request.
